# The effects of humic water on endothelial cells under hyperglycemic conditions: inflammation-associated parameters

**DOI:** 10.1007/s10653-018-0238-1

**Published:** 2019-01-04

**Authors:** Katarzyna Szot, Krzysztof Góralczyk, Małgorzata Michalska, Natallia Veryho, Jacek Chojnowski, Irena Ponikowska, Danuta Rość

**Affiliations:** 1 Uniwersytet Mikolaja Kopernika Collegium Medicum, Bydgoszcz, Poland; 2 Kujawska Szkoła Wyższa, Włocławek, Poland

**Keywords:** Humic water, Endothelial cells, HUVEC, Hyperglycemia, Inflammation

## Abstract

Humic waters (HW) are globally unique, deep underground, dark-brown waters containing humic acids, and they present numerous therapeutic activities including anti-inflammatory. In the present study, we use HW from source in Poland. Diabetes has become an epidemic and is a risk factor of cardiovascular diseases. Hyperglycemia in diabetes is responsible for damaging of the endothelium and increases inflammation on the surface of the vascular lining. The inflammatory process in diabetes is associated with the secretion of inflammatory cytokines by endothelial cells, e.g., tumor necrosis factor alpha (TNFα) and interleukin 6 (IL-6), and with the reduction of cell proliferation. In the study, we used cultures of endothelial cells (HUVEC line—human umbilical vein endothelial cells) with the addition 30 mM/L of glucose in the culture medium which imitated the conditions of uncontrolled diabetes. The addition of HW in the proper volume to the culture medium causes reduction of inflammation by significant decrease in inflammatory cytokines such as TNFα and IL-6 and also leads to enhancement of the cell proliferation. It appears that the adverse effects of hyperglycemia on vascular endothelial cells may be corrected by addition of humic water. The above promising results of in vitro tests provide an opportunity to the possible use of humic water in the supportive treatment of endothelial dysfunction disorders in diabetes. However, this issue requires further clinical research.

## Introduction

Humic waters (HW) are globally unique, deep underground, dark-brown waters containing humic acids, found in only a few countries in Europe. In Poland, HW from the water-bearing layers (the oldest Neogene Period) of the Miocene occur in the Wielkopolskie Voivodeship, near Poznań, where 16 sources, including a source in Brączewo, have been described and studied. Humic waters are a natural extract of humic substances (mainly humic and hymatomelanic acids) which give them a specific color. These acids are polymers consisting of an aromatic core of amino acids, sugars, peptides and other aliphatic components. They are decomposition products of organic matter of plant origin, formed during a process called humification (Latour et al. 2015). Humic acids have numerous biological and therapeutic effects (Van Rensburg 2015; Rupiasih and Pandit 2008; Kloecking and Helbig 2005). Biochemical and bacteriological analyses of HW have shown that these waters are originally chemically and bacteriologically pure, physically and chemically stable, and could be used as drinking waters (Latour et al. 2015).

Diabetes has become an epidemic and is projected by the WHO to be the seventh leading cause of death worldwide by 2030. Despite significant advances in treatment, complications of this disease are still a significant clinical problem, as the condition of good metabolic control does not fully protect against the development of complications related to pathological changes in the vascular endothelium in diabetic patients. Among the complications of diabetes, the most important is damage to large vessels (macroangiopathy) and small vessels (microangiopathy), because disorders of endothelial function and structure initiate these changes. The vascular endothelium, which is more and more often seen as the largest endocrine gland in the human body, is an important element combining cellular disorders and clinical effects. Hyperglycemia itself affects the activation and stimulation of endothelial cells, leading to disruption of the multifactorial balance provided by these cells (Schalkwijk and Stehouwer 2005). Under hyperglycemic conditions, excessive production of reactive oxygen species (ROS) is one of the key factors in the development of pathological changes in the endothelium; the endothelium is characterized by changes in proliferation, and the apoptosis process is intensified. Endothelial pathology is accompanied by excessive production of factors, called endothelial dysfunction markers, related to the inflammatory process, such as tumor necrosis factor—TNFα—or interleukin 6 (IL-6) (Pedersen 2007).

In the study, we used cultures of endothelial cells with the addition of 30 mM/L glucose in the culture medium. The similar methods of cultures have been previously used to investigate hyperglycemic conditions experimentally (Góralczyk et al. 2016).

## Materials and methods

The approval of Bioethics Commission of the Nicolaus Copernicus University, Collegium Medicum in Bydgoszcz, was obtained. Reference number: KB/135/2009.

### Human umbilical vein endothelial cells (HUVEC) isolation and culture

Endothelial cells (HUVEC line) were derived from human umbilical veins by the enzyme method using collagenase (Jaffe et al. 1973). Cells were cultured in M199 media supplemented with 20% fetal bovine serum (FBS), 100 U/mL penicillin (Gibco^®^ products), and growth factors 50 μg/mL endothelial cell growth supplement (ECGS-Corning Inc. USA) and heparin. The cells were incubated at 37 °C in humidified atmosphere with 5% CO_2_. After two to four passages and seeding of the cells in 6-well culture plates, the proper experiment was conducted. The experiment was repeated three times with three independent cells isolations.

To imitate hyperglycemic condition, 30 mM/L glucose was added to the culture medium. The purpose of adding glucose was to create conditions similar to those that can be met in blood vessels of diabetic patients. In the study groups 2 and 4, an appropriate volume of humic water was added to obtain its 1% solution in the culture medium.

In our study, we used the humic water from a spring in Brączewo, and it is originally bacteriologically pure, physically and chemically stable, bicarbonate–chloride–sodium water containing 192.2 mg/dm^3^ of humic acids (Latour et al. 2015). The water content of cations, anions and specific therapeutic compounds is presented in Table 1.

**Table 1:** Content of cations, anions and specific therapeutic compounds in humic water

Parameter	Concentration (mg/dm^3^)
Na^+^	406.5
Ca^2+^	27.1
Mg^2+^	9.11
Cl^−^	407.7
HCO^3−^	520.8
SO^4−^	< 1.0
K^+^	6.14
Fe^2+/3+^	2.15
F^−^	1.41
J^−^	0.09
H_2_SiO_3_	14.2
Humic acids	192.2
Mineralization	1395.2

The study was conducted in four groups (Table 2): group 1—no glucose in culture medium, no humic water (control group); group 2—no glucose, humic water added; group 3—added glucose in culture medium, no humic water; and group 4—added glucose, humic water in culture medium.

**Table 2:** Results of the tested parameters in cell culture research groups

	1 Group	2 Group	3 Group	4 Group	
	Control group	+1% HW	+30 mM/L glucose	+ 30 mM/L glucose + 1% HW	
Number of cell (N × 10^5^ cells)					
M	4.2	4.4	3.4	4.2	0.0040*
SD	0.6	0.8	0.9	0.9	
TNFα (pg/10^5^ cells)					
Me	1.6	1.9	2.5	1.7	0.0489*
IQR	1.3–2.0	1.4–2.8	1.5–3.6	0.9–2.3	
IL-6 (pg/10^5^ cells)					
Me	41.7	43.8	56.2	39.9	0.0229*
IQR	35.0–47.8	37.5–60.5	39.8–75.8	28.4–54.5	

*HW* humic water, *M* mean, *SD* standard deviation, *Me* median, *IQR* interquartile range

*P value—group 3 versus 4, group 1 versus 2—not statistically significant

### Cell counting with the use of trypan blue dye

For evaluation of endothelial cells number, the cells on the bottom of each well were harvested by using trypsin and counted by hemocytometer. This method uses trypan blue dye according to the method described by Basso et al. (2013). The trypsinized cell suspension was mixed with the equal volume of 0.4% trypan blue. Ten microliters of the solution was taken to a hemocytometer and examined with an inverted light microscope (Nikon Eclipse TE2000-S). Cell viability was determined by trypan blue exclusion test and was > 95% in all experimental groups.

### Measurement and analysis of TNFα and IL-6

At the end of experiment, conditioned medium from each well of culture plates was collected and frozen at − 86 °C. After thawing, the concentration of TNFα and IL-6 in the supernatant was measured by ELISA test (Diaclone) according to the manufacturer’s instructions. The results of the parameters concentration in the supernatant from each well of culture plates were analyzed per number of cells in each suitable well.

### Statistical evaluation

Statistical analysis was performed using Statistica 13.1 (Dell Inc.). The t-Student test was used for parametrical analysis (number of cell), and Mann–Whitney nonparametric test was used for comparisons of TNFα and IL-6. Statistical significance was defined as *P* < 0.05. The results are presented as mean (M) ± standard deviation (SD) or median (Me) and interquartile range (IQR).

## Results

The results obtained in the study are presented in Table 2. The number of HUVECs was higher in group 2 (humic water), and slightly lower in control group. The lowest number was observed in group 3, cultured under hyperglycemic conditions, while the number of cells in group 4 with glucose and humic water reached the level similar to the control group. A significantly higher amount of endothelial cells was noted in the group 4 compared to group 3 consisted of endothelial cells in 30 mM/L glucose conditions, and the difference in relation to the group 3 was statistically significant (*P* = 0.004).

The concentration of TNFα and IL-6 increased after the addition of glucose to the culture medium in group 3. Adding humic water to the culture medium with glucose causes decrease in TNFα and IL-6 level in group 4 to the level similar to the control group and group with humic water. The differences between groups 3 and 4 for TNFα (*P* = 0.0489) and IL-6 (*P* = 0.0229) were statistically significant.

## Discussion

Despite the fact that humic acids contained in organic-mineral peloids have been successfully used for a long time in the treatment of many diseases related to inflammatory aetiology, the knowledge about their biological effects on the cellular level is rather limited. Most studies concern local application of products containing humic acids (Vysokogorskii et al. 2009). An additional limitation in the comparison of the anti-inflammatory properties of humic substances is the use of various extracts of humic acids in various forms, coming from many sources naturally occurring in different latitudes, as well as from a chemical synthesis (Wolina 2009; Snyman et al. 2002). The aim of this study was to determine the effects of humic water from a source in Brączewo as an effective agent that could be used to counteract the undesirable effects of hyperglycemia on the initiated inflammatory process. The unquestionable advantage for the application of HW is its original bacteriological purity and chemical and physical stability in a time, resulting from the underground location of the water sources, without any effects of atmospheric and anthropological factors (Latour et al. 2015).

Since cardiovascular risk burden is not eradicated by intensive glycemic control, cellular mechanism-based therapeutic strategies are highly desirable. In vitro cultures of endothelial cells, despite their limitations such as no interactions with the microenvironment and other cells, the absence of physical forces (shear stress), seem to be reliable models for studies (Kvietys and Granger 1997).

In this study, the number of endothelial cells obtained in culture was lower in the group with glucose added, i.e., under conditions imitating the endothelial environment in diabetes. The addition of humic water resulted in an increase in the number of cells to a number comparable to that obtained in the control group, similar to the endothelium functioning normally in the vessels (Table 2). We have, therefore, obtained evidence that the adverse effects of hyperglycemia on endothelial cells could be corrected by the addition of humic water. Humic substances modulate the activity of ion channels associated with membranes and may contribute to restoring normal proliferation of endothelial cells, despite negative effects of hyperglycemia. The results obtained in the study regarding the effects of LLLT on endothelial cells in the same culture model, as compared to the results obtained with the use of HW, showed advantages of humic water with a positive effect on the normalization of the studied parameters.

High glucose levels disrupt the normal oxidation–reduction reaction of the endothelial cells, inducing oxidative stress (Baynes 1991). Humic water has oxidizing–reducing properties and can act as oxidizers or reducers, i.e., they are capable of producing as well as binding reactive oxygen species. Oxidation and reduction of humic substances are reversible, reproducible process. These properties may explain normal proliferation of endothelial cells despite the artificially formed hyperglycemic state in the study.

Hyperglycemia is considered to be the primary factor responsible for endothelial dysfunction expressed by increased levels of markers of the inflammatory process released from these cells. Interleukin 6 (IL-6) and TNFα (tumor necrosis factor) are typical examples of multifunctional cytokines secreted by endothelial cells involved in inflammation. Increased secretion of TNFα and IL-6 under hyperglycemic conditions has been observed in many studies (Liu et al. 2013; Wang et al. 2012). Hyperglycemia induces the production of proinflammatory cytokines and growth factors by activating key signal pathways associated with MAPK (mitogen-activated protein kinases), NF-κB (nuclear factor κB) and STAT3 (signal transducers and activators of transcription depending on ROS and oxidative stress) (Sweet et al. 2009). This is due to the effect of TNFα on the modification of a redox reaction in the mitochondrial respiratory chain (Mariappan et al. 2007; Suematsu et al. 2003).

The analysis of levels of markers of the inflammatory process, secreted by endothelial cells in individual study groups, shows that humic water has a beneficial effect on the reduction of inflammation induced by hyperglycemia. The addition of humic water to a culture medium resulted in decreased TNFα and IL-6 secretion in comparison with cells exposed only to glucose (Table 2). Van Rensburg and Naude (2009) have also shown that preparations containing humic substances inhibit the release of cytokines associated with inflammation under in vitro conditions. Similar results were obtained by Junec et al. (2009) who used preparations containing humic acids at concentrations > 200 μg/mL in the neutrophil tests under in vitro conditions. On the other hand, Chen et al. (2002) indicated that humic substances stimulated the release of proinflammatory cytokines, such as TNFα in vitro, but only in the presence of exogenous lipopolysaccharides. This means that these humic substances do not seem to cause inflammation under normal conditions (a slight increase in TNFα and IL-6 secretion in the control group with the addition of HW), and the greatest benefits are derived from the use of HW in pathological conditions with a deficit in oxygen metabolism.

Spilioti et al. (2017) studied therapeutic properties of mud wraps containing humic acids used in various spa resorts. Significant reduction in the secretion of inflammatory factors at the cellular level has been observed. Humic water has a “buffering effect,” which means that they are able to produce and bind ROS. This is in line with the view that humic substances have a great potential as natural antioxidant. This regulatory system is believed to be important for the beneficial effect of humic water on the endothelium in diabetic patients. Cultured endothelial cells are useful addition to in vivo studies for defining the beneficial effect of HW. The possible therapeutic use of humic water per os requires further basic studies, followed by animal models and ultimately clinical trials.

## Conclusions

1. The effects of hyperglycemia on endothelial cells, which reduce the viability of the culture, can be corrected by the addition of humic water. The beneficial effects of HW are expressed by restoring the correct number of endothelial cells and decreasing the inflammatory process intensity (based on the IL-6 and TNFα levels) caused by high glucose concentrations in the culture medium.
2. Humic water does not affect the vascular endothelium, which was confirmed in laboratory conditions.
